# Tau-protein concentrations are not elevated in cerebrospinal fluid of patients with progressive multifocal leukoencephalopathy

**DOI:** 10.1186/s12987-019-0148-3

**Published:** 2019-09-05

**Authors:** Nora Möhn, Yi Luo, Thomas Skripuletz, Philipp Schwenkenbecher, Inga Zerr, Peter Lange, Martin Stangel

**Affiliations:** 1 Department of Neurology, Hannover Medical School, Carl-Neuberg-Str. 1, 30625 Hannover, Germany; 2 Neurochemical Laboratory, Department of Neurology, University Medicine Göttingen, Robert-Koch-Str. 40, 37075 Göttingen, Germany

**Keywords:** Progressive multifocal leukoencephalopathy, Tau-protein, Biomarker, Cerebrospinal fluid, Immunosuppression

## Abstract

Progressive multifocal leukoencephalopathy (PML), caused by infection with John Cunningham polyoma virus (JCPyV) in immune-compromised patients, is a serious demyelinating disease of the central nervous system. This disease often leads to major neurological impairments and consecutive disability. No effective treatment for PML has been found as yet. As JCPyV-PCR of the cerebrospinal fluid (CSF) may be negative in some cases, a reliable diagnosis might prove to be difficult as well. So far, two case reports suggested CSF-tau to be a promising biomarker for PML. Our study included 10 patients with assured diagnosis of PML and varying underlying diseases. In all but one the CSF-tau concentration was normal. Our results indicate that CSF-tau is not an appropriate biomarker for PML.

## Introduction

PML is a central nervous system (CNS) demyelinating disease induced by the infection with John Cunningham polyoma virus (JCPyV). PML mostly occurs in immune-compromised patients, for example with acquired immune deficiency syndrome (80%), hematologic malignancies (13%), organ transplant recipients (5%), and patients treated with immunosuppressive drugs (3%) [1, 2]. The disease is rare, but is characterized by high mortality rates and long-term neurologic morbidity. The diagnosis of PML is generally based on clinical, imaging, laboratory, and histopathological features. The detection of JCPyV DNA in CSF in combination with appropriate clinical symptoms, and radiological characteristics contributes to a definite diagnosis of PML. In individual cases the presence of characteristic pathoanatomic findings from a CNS biopsy specimen may be required to establish the correct diagnosis [3]. Undoubtfully, an early diagnosis and quick therapeutic approach to the immune deficiency could improve the prognosis. Reasons for a delayed diagnosis of PML may be (1) the diversity of its clinical symptoms depending on the lesion localization, (2) the lack of typical radiographic features in brain magnetic resonance imaging (MRI), and (3) the fact that JCPyV-PCR may be negative, especially at an early stage of disease. In case of clinical suspicion, an invasive brain biopsy might be required. Consequently, identification of potential biomarkers for PML is a clinical need for patients with clinical suspicion and negative CSF PCR.

Tau is a microtubule-associated protein predominantly expressed in CNS neurons and has been verified as an important factor in the formation and subsequent stabilization of microtubules, as well as for the organelle movement of axons and dendrites [4]. In recent years, immunohistochemical studies indicated that neuroaxonal degeneration and neurological inflammation may result in an increased release of tau from neurons and an enhanced phosphorylation of tau. Notably, tau protein in CSF has been recognized as an important biomarker for degenerative CNS diseases like Alzheimer’s disease [5]. Besides neurodegenerative diseases some reports also suggest that the CSF concentrations of total tau (t-tau) and phosphorylated tau (p-tau) are different between patients with viral or bacterial neurological infections compared with healthy individuals [6].

There is one case report which suggested that an elevated CSF t-tau may be a biomarker for PML. In this case of a patient with B-cell lymphoma associated PML t-tau was excessively elevated in the CSF while p-tau was rather low [7]. Another study, investigating CSF tau-concentrations in HIV patients, included one HIV+ patient with diagnosed PML who exhibited an elevated CSF tau-concentration. The authors of the study observed an association between elevated CSF tau and poor survival in HIV+ patients, but also pointed out that CSF tau was not increased in the majority of patients [8]. So far, there was no systematic investigation of t-tau in the CSF of PML patients. We therefore studied t-tau and p-tau concentrations in patients with PML and different underlying conditions leading to immunodeficiency.

## Methods

### Measurement of CSF tau-concentrations

All patients were treated at the Department of Neurology at Hannover Medical School. CSF samples were obtained via lumbar puncture in sitting position. After a centrifugation process all samples were frozen in aliquots within 2 h after lumbar puncture and stored at − 80 °C until analysis. Tau- and p-tau-concentrations within the CSF were measured via solid-phase enzyme-linked immunosorbent assay (ELISA). For all analyses the ELISA-Kits INNOTEST hTAU Ag and INNOTEST PHOSPHO-TAU (INNOGENETICS, Heiden) were used and all measurements were performed according to the laboratory standards of the neurochemical laboratory at the University of Göttingen. After a coating-step with a monoclonal antibody (AT120 or HT7, respectively) CSF-samples were added. Following several washing steps, samples were incubated with biotinylated antibodies (HT7 and BT2 or AT270bio, respectively). Antigen–antibody complexes were detected via color reaction between peroxidase-labelled streptavidin and a substrate solution. The reference range for CSF tau-concentrations is 80–450 pg/ml. For CSF p-tau-concentrations, a maximum of 60 pg/ml is considered normal. Reference values are based on the 2001 publication by Sjögren and colleagues, who analyzed Tau-concentrations in CSF of 231 neurologically and psychiatrically healthy individuals aged between 21 and 93 years [9]. Furthermore, the local laboratory reference was considered as well.

The study was approved by the local institutional review board (Hannover Medical School, 2413-2014).

### Statistical analysis

The continuous variables are represented as mean ± SD. The association between concentrations of t-tau, p-tau and age, modified Rankin Scale (mRS) score, and the copy number of JCPyV DNA in PML patients was assessed using correlation analyses. Values of P < 0.05 were considered statistically significant. All statistical analyses were performed using the SPSS program (Version 24.0, IBM Statistics).

## Results

### Patients characteristics

A total of 10 patients were enrolled in the present study. Their baseline characteristics are summarized in Table 1. Except of one patient, all were male with a median age of 56 years (range 31–77 years). The cohort contained three patients with HIV infection, four suffered from neoplastic or myeloproliferative diseases, two patients with multiple sclerosis (MS) received natalizumab, and one patient was immunosuppressed with tacrolimus and mycophenolate mofetil due to liver transplant. JCPyV DNA was detectable via polymerase chain reaction (PCR) in CSF of 9 patients with values ranging from < 180 to 200,000 copies/ml. In one patient PML was detected via stereotactic biopsy of the brain.

**Table 1:** Demographics and clinical characteristics of PML patients

Case	P1	P2	P3	P4	P5	P6	P7	P8	P9	P10
Age (years)	77	76	55	43	72	31	57	54	54	42
Sex (M = male F = female)	M	M	M	M	M	M	M	M	M	F
UID	BC	NHL	CLL	MS	LT	HIV	HIV	MM	HIV	MS
mRS after PML	2	4	5	3	2	2	3	2	3	4
JCPyV DNA (c/ml)	100.000	200.000	499	500	Neg. (brain biopsy)	1080	800	179	249	249
T-tau (pg/ml)	332	374	218	190	121	568	87	239	389	118
P-tau (pg/ml)	49	34	24	43	36	62	22	51	56	36

*UID* underlying immunodeficiency, *mRS* modified Rankin Scale, *BC* bronchial carcinoma, *NHL* non-Hodgkin lymphoma, *CLL* chronic lymphocytic leukemia, *MS* multiple sclerosis treated with natalizumab, *LT* liver transplant, *MM* multiple myeloma, *HIV* human immunodeficiency virus

### Tau concentrations in CSF of PML patients

The CSF t-tau levels were 270.6  ± 145.3 pg/ml (mean ± SD) and the CSF p-tau levels were 41.3 ± 13.3 pg/ml in patients with PML. In 1 of the 10 patients, the CSF t-tau level was slightly increased (568 pg/ml), whereas all other values were within normal range (< 450 pg/ml). All patients exhibited normal CSF p-tau concentrations (normal range: < 61 pg/ml). Due to the lack of a control group, CSF tau concentrations of our cohort have been compared with commonly accepted reference values (see “Methods”).

There was no correlation of CSF t-tau or p-tau concentrations with age, mRS score, or the copy number of JCPyV DNA in PML patients (Table 2).

**Table 2:** Correlation analysis with t-tau and p-tau

	Age	MRS	JCPyV DNA
t-tau			
Correlation coefficient	− 0.146	− 0.342	− 0.108
p	0.688	0.333	0.799
p-tau			
Correlation coefficient	− 0.451	0.143	− 0.096
p	0.191	0.694	0.821

## Discussion

One previously published case report suggested that the level of t-tau protein may be increased in the CSF of PML patients who suffer from B-cell lymphoma [7]. The authors implied that this might be a novel promising biomarker for the diagnosis of PML. To clarify this hypothesis, 10 PML patients were included in our study to measure the concentrations of t-tau and p-tau in CSF via enzyme-linked immunosorbent assay. It is noteworthy that the t-tau levels were normal in 9 of 10 patients and p-tau concentrations were normal in all patients. Their values did not correlate with age, JCPyV DNA in CSF, or severity of the disease. As PCR-negative PML is not very common only one PML-patient in our series was JCPyV-negative. Thus, a statistical comparison between JCPyV-negative and JCPyV-positive patients was not feasible. Compared with reference values of healthy individuals CSF tau-concentrations were not elevated in our PML patients regardless of their JCPyV-status. It seems extremely unlikely that PCR-negative PML patients will have other CSF tau/p-tau concentrations. Collectively, our data suggest that neither t-tau nor p-tau is a predictive biomarker for PML independent of the patient’s JCPyV-status.

Our findings contradict the previously mentioned case report that showed increased t-tau concentrations in CSF of a PML patient, and was supported by studies which suggested that axons may be injured or damaged by the loss of myelin protection [10]. In contrast to the previously published case report, we included more CSF samples from PML patients with different underlying diseases. It remains unclear why the t-tau concentrations were elevated in the previously published cases. We only can speculate that the patients suffered of unknown comorbidities.

This study has several limitations: first, this work is based on data from a single institution. Secondly, the number of analyzed CSF specimen is low, although this is the first study which measured CSF-tau concentrations in more than one PML patient. Nevertheless, our results are so clear-cut that investigation of more samples is not necessary.

In conclusion, CSF-tau protein does not meet the rigorous standards for a clinical biomarker for PML.

## Conclusion

PML is a threatening disease in which early diagnosis is highly relevant. As JCPyV-PCR may be negative there is need for a reliable biomarker. We examined CSF-tau protein in a cohort of 10 PML positive patients. In conclusion, we can state that CSF-tau protein does not meet the rigorous standards for a clinical biomarker for PML.

## Data Availability

All data generated or analyzed during this study are included in this published article.

## Abbreviations

PML: progressive multifocal leukoencephalopathy
JCPyV: John Cunningham polyoma virus
CSF: cerebrospinal fluid
CNS: central nervous system
MRI: magnetic resonance imaging
t-tau: total tau
p-tau: phospho-tau
ELISA: enzyme-linked immunosorbent assay
mRS: modified Rankin Scale
MS: multiple sclerosis
PCR: polymerase chain reaction
